# Viapolitics and the emancipatory possibilities of abortion mobilities

**DOI:** 10.1080/17450101.2020.1803588

**Published:** 2020-08-06

**Authors:** Cordelia Freeman

**Affiliations:** Department of Geography, University of Exeter, Exeter, UK

**Keywords:** Abortion, mobility, reproductive health, transport, travel, viapolitics

## Abstract

Scholarship on abortion travel has examined the places women travel between and why such journeys are necessary. However, there has been scant attention paid to the journeys themselves and *how* these journeys are undertaken. This paper uses William Walters’ notion of ‘viapolitics’ to better attend to *how* people travel by focussing on the role of vehicles in abortion politics. This takes three parts: an exploration of the emotional and embodied journeys that women have to take to access abortions; the role of the vehicle as a site of political activism around abortion rights; and the transportation of abortion medication. Viapolitics has to date only been used within migration politics but as this paper shows, it has utility beyond this field to interrogate abortion travels and highlight the role of vehicles in abortion access as well as to explore how abortion transport can be emancipatory for women. This paper furthers viapolitics by arguing that we need to consider the journeys of ‘things’ and not just people. In the case of abortion access, it is the transportation of abortion medication rather than the travel of women that is the most socially just solution to discriminatory laws and extra-legal barriers.

## Introduction

A young woman sits across from me and recalls her decision to undertake the journey from her home in Chile to a clinic in Peru for an abortion. She was fortunate; her family supported her, the journey only took an hour and a half, and her aunt recommended a clinic. But before and during the journey she was terrified. The procedure was illegal in both Chile and Peru, and she could face prison if caught. She also didn’t know what would happen to her body or how to manage the bleeding as she travelled back home.

The ‘new mobilities paradigm’ has stimulated and advanced critical analyses of movement and stillness, but not all types of movement or groups of people have been fully included. Reproduction and gender have been two gaps in the mobilities literature. In this paper I propose ‘abortion mobilities’ as a way to rectify these gaps. Here I define abortion mobilities as the movement and fixity of people and things that shape abortion access. I make this contribution by building upon the burgeoning scholarship of ‘reproductive mobilities’ through showing how William Walters’ (2015a) concept of ‘viapolitics’ can offer a critical perspective on abortion (im)mobilities, and vice versa. I argue that this provides a way to explore *how* journeys for abortion access are undertaken and provides a framework of analysis for the embodied and emotional experience of the above woman who embarked on such a journey. Furthermore, this paper make a case for the introduction of viapolitics into mobility studies and shows how it can be applied and advanced.

Mobilities and reproduction have been ‘mostly disparate bodies of scholarship’ (Speier, Lozanski, and Frohlick 2020, 108) even though ‘[m]obilities theory can offer many new insights into reproduction, while feminist and critical theories of reproduction might allow for new insights into theorizing (im)mobilities’ (Sheller 2020, 188). The majority of scholarship on reproductive mobility has focused on transnational travel for in vitro fertilization (Bergmann 2011) and surrogacy (Deomampo 2013). Thus, there has been a disproportionate focus on ‘procreative tourism’ (Inhorn and Patrizio 2012) as opposed to abortion. Alongside reproduction, gender also remains understudied in the mobilities literature. Gender is now more included in analyses of mobility than ever before (see Uteng and Cresswell 2008; Yeoh and Ramdas 2014), yet according to Clarsen (2014, 95) ‘the radical potential of the concept of gender has not been exhausted in mobilities scholarship and, conversely, a broad mobilities paradigm has not had a significant impact on feminist theorising’. This paper responds to such critiques by bringing together critical analyses of mobilities research and feminist theories of gender and reproduction. Mobilities scholarship needs to be better attuned to embodiment and emotion (Fortunati and Taipale 2017), and so this paper takes a particular focus on bodies and emotion in abortion (im)mobilities.

Mobility is so often political, as apparent through who gets to move, how fast people get to move, what route is taken, how mobilities are represented, and whether mobility is chosen or forced (Cresswell 2010). This is all true of abortion access. Abortion journeys vary enormously depending on who is taking it and in what context. Some will have a relatively short journey (Pheterson and Azize 2005) whereas others will take long, expensive, illegalised journeys in immense pain. These diverse experiences are dictated by myriad factors such as class, citizenship, ethnicity, and cultural context. Abortion access is far from equal and while some women are forced to travel long distances, others are not, or cannot. The politics of mobility thereby must contemplate both movement and stasis (Hannam, Sheller, and Urry 2006). Abortion travel is not new; women have been traveling internationally ever since some countries liberalized abortion in the 1960s, but abortion access is changing with the use of abortion medication. Pills now move across the globe and so we need a framework that includes things as well as people. Pruitt and Vanegas (2015, 86) noted that abortion access is ‘an acutely spatial phenomenon’ but as I argue here it also acutely concerns (im)mobility, making it a productive research frontier for the growing mobilities research agenda. The following section explains how ‘viapolitics’ is well placed to do this critical, political work and how I use it to further abortion mobilities.

## Travelling with viapolitics

William Walters (2015a) coined the term ‘viapolitics’ to provide a framework for centring vehicles, roads, and routes in theorising migration politics from the perspective of the in-between. He argues that these are not entirely absent from migration and border studies, ‘[b]ut, with certain notable exceptions … they rarely feature as a central focus in theorization and investigation of migration worlds’ (2015a, 470). Walters argues that viapolitics shifts how migration can be viewed from the angle of the vehicle rather than the state, and shows that vehicles are sites of power and contestation in their own right. This focus on vehicles is used by Walters to show how they themselves can become a site of strategic political action. For Walters, vehicles need to receive more serious attention and through his work on deportation flights he has shown how the aeroplane is entangled in struggles over power and injustice (Walters 2016).

Viapolitics has been taken up by other scholars but only within work on migration. Teunissen (2018, 1) used viapolitics in his analysis of mobility in Europe through the case study of German coach operator FlixBus and agreed with Walters’ argument that this focus on vehicles allows for ‘a more theoretical and comprehensive understanding of human mobility and its entanglement with power relations’. It has also been used by Senu (Forthcoming) in his work on stowaways in the global shipping industry to centre the cargo ship as a site of governance and contestation, identity, and community and by Bialasiewicz and Maessen (2018) to make sense of the centrality of transport in the movement of refugees at the boundaries of the EU. Likewise, in their study of European migration, Heller, Pezzani, and Stierl (2017) briefly reference Walters and the utility of viapolitics in understanding the migrant boat as a site of political action. While still a relatively new concept, viapolitics has, then, been utilised by researchers working on migration politics. This paper is the first to take viapolitics outside of migration scholarship and, in using it as a framework to understand abortion mobilities, I will show that the concept has utility beyond the arena in which Walters conceptualised it and can enrich mobilities scholarship.

Given that viapolitics has only been conceptualised within work on migration, it has yet to be brought into direct conversation with mobilities scholarship more broadly. There is rich potential here, particularly given a broad range of literature on transport. Viapolitics does bring transport into its analysis but it is doing something different. The ‘via’ that is ‘political’ refers not to a singular mode of transport, infrastructure, or node, but in how movement brings these (and others) together with political ramifications. This sustained critical analysis and insistence that transport cannot be understood in an isolated way means that Walters’ (2015a) contribution has revealed the politicised nature of journeys and vehicles, and the power relations that politicise them. To date, viapolitics has not been taken up in a major way in mobilities scholarship and the term has never appeared in this journal. This paper will illustrate how viapolitics makes a useful contribution to critical mobilities scholarship and will do this through my work on abortion mobilities.

The concept of viapolitics furthers abortion mobilities specifically through an interrogation of how and why the actual travel and transport of women and abortion medication matters. I encourage researchers to examine not just that people are forced to travel and the reasons for this, but *how* they travel. Viapolitics is well situated for this task because by centring vehicles, roads, and routes, the frame of study can be shifted away from borders and the state. This resonates with the argument put forward by Calkin (2019) that abortion scholarship needs to move beyond the state as abortion politics are multi-scalar, entangling sub-state regulations and transnational technologies. I take the notion of viapolitics and the figure of the vehicle as a way to cut through scale and understand abortion from the scale of the body to the global. This approach illustrates how viapolitics can help us to analyse abortion journeys and thereby understand abortion politics in new ways. By taking vehicles, roads, and routes more seriously in scholarship on abortion access, this paper will illustrate how journeys are entangled with the politics of abortion provision and prohibition and will stretch viapolitics beyond its original conception in migration politics.

This paper will propose ‘viapolitics’ (Walters 2015a) as a useful analytical framework to interrogate and foster ‘abortion mobilities’. In doing so it makes three theoretical contributions: it furthers the idea of ‘abortion mobilities’; brings the concept of viapolitics into mobilities scholarship; and proposes the term ‘emancipatory viapolitics’. I ground this theoretical work in my own empirical research in Chile, Peru, and Mexico as well as by drawing on secondary sources from Europe and North America. The structure of this paper follows the three ways in which viapolitics can be fruitfully utilised to analyse the politics of abortion mobilities. The first relates to the embodied and emotional aspects of the abortion journey with a specific focus on bleeding in vehicles and the politics of fear. The second aspect politicises vehicles to show how they are sites of political resistance around abortion access that generate powerful visual media. The third and final way is to argue that we need to focus more on what is doing the travelling, in this case it is the transport of abortion medication that can be the most emancipatory possibility by taking away the need for abortion travel.

## The abortion journey

Global abortion travel really began in the 1960s when certain countries such as Japan, Poland, Sweden, Mexico, and Switzerland relaxed their abortion laws and became destinations for women who were able to travel (Sethna and Doull 2012). The ability to travel and the experiences of that travel remains highly asymmetrical with young women, indigenous women, rural women, women on low incomes, and trans and non-binary people disproportionately affected by obstacles to abortion access (Doran and Nancarrow 2015; Silva and McNeill 2008). Logistical issues such as childcare, accommodation, transportation costs, and taking time off work compound these intersectional issues to further prohibit access (Doran and Hornibrook 2016). As Jerman et al. (2017) importantly emphasize, these barriers do not work in isolation; the negative impacts increase when multiple barriers are experienced. Each abortion journey is different. These differences are informed by structural issues as well as more individual ones such as a woman’s particular childcare arrangements or access to transport. These factors intersect to create specific experiences of oppression.

Scholarship on abortion travel has examined the locations women travel between in order to procure an abortion and why such journeys are necessary (Sethna and Davis 2019; Doran and Nancarrow 2015). Such research has highlighted the obstacles to abortion access posed by poor public transport infrastructure and the challenges of private transport, particularly for those in rural areas (Brown 2019; Gomez 2016; Marty 2019; Statz and Pruitt 2019). This is important work, but it tends to lack a sustained focus on the embodied and emotional experiences of women *during* the abortion journey. In this section I illustrate how the politics of, firstly, blood and, secondly, fear during abortion travel prompts us to heed Walters’ (2015a) argument that ‘it is time to take vehicles more seriously’. In focusing on bleeding and fear, it becomes clear why abortion is a political issue and why it is important to understand how abortion travel is experienced.

Vaginal bleeding is common after both surgical and medical abortions, usually lasting one to two weeks, but in some cases up to a month after a medical abortion.^1^ Through my research on abortion access in Latin America the politics of blood was a recurring theme. In Mexico, abortion has been legal in Mexico City since 2007 and in late 2019 was legalised in the state of Oaxaca. In the rest of Mexico abortion is illegal with some state -dependent specific rules that allow for it while other states even criminalize miscarriages. This legal patchwork means that women travel to Mexico City to access legal abortions, primarily from the states nearest the capital. Having to travel impacts the type of abortion women seek. I interviewed doctors in Mexico City who said that many women who travel choose a surgical abortion over a medical one as the bleeding tends to be easier to hide so the abortion can be kept a secret from friends and family and the woman will know with absolute certainty that the procedure was successful. This is particularly appealing for women who have to travel after their abortion. Travelling home by plane, bus, or car knowing that the abortion has taken place and that the bleeding should be of a shorter duration eases the journey.

The fact of bleeding also makes women vulnerable to the risk of being caught in jurisdictions where the practice is illegal. As my research on women who travel from northern Chile to southern Peru for abortions has shown, women still face legal penalties even if the abortion took place outside of Chile (Freeman 2017, 2015). In 2013, a 25 year old woman had travelled from Arica in northern Chile to the Peruvian city of Tacna where abortion medication is much easier and cheaper to obtain. She took the medication in Tacna but due to its delayed effect she was bleeding as she returned to Arica. The abortion was incomplete and so she was admitted to hospital where she disclosed what she had done. Thus, blood and bleeding en route becomes an incriminating marker.

Testimonies of bleeding on vehicles have also been successful at changing legislation. Personal stories of what women have been through bring the embodied and emotional dimensions of abortion travels to the fore and help us better understand the abortion journey. Under English law until 2018, women had to take the medication in a healthcare setting, meaning that the abortion often started as they returned home. Claudia Craig, 23, a recent graduate who had a medical abortion in England at seven weeks publicly shared her traumatic experience of bleeding in a taxi and struggling with cramps and nausea in order to contest this law. As she wrote in one article ‘[e]ach woman’s experience of this will differ, but for me it involved feeling extremely faint, cramping, vomiting, and significant bleeding. It also started in a taxi’ (Craig 2018, np). Craig told one publication that if she had been allowed to take the second pill at home ‘I wouldn’t have been rushed and panicked and worried about going through an abortion in a taxi, and I could have taken the pill in the place and time that was right for me’ (Apostolides 2018, np). Another woman, using the pseudonym Paula, stated that after taking the second dose of the medication in a clinic an hour away from her home ‘I began to miscarry on the way home. I just wanted to be in bed, not in a car on a motorway, as I started to feel ill really quickly’ (BBC News 2018, np). Legal particularities that forced women to take the medication in clinics created the scenario where women were forced to bleed in vehicles.

Partly due to the sharing of these experiences, the law has changed. Women in Scotland have been permitted to take the medication in their own homes since 2017, in Wales since June 2018, and in England since December 2018. Abortion only became legal in Northern Ireland in October 2019 and to date the medication still cannot be taken at home. Indeed, despite the legal changes on where medication can be taken, women who travel from Northern Ireland to England for abortions are still discriminated against as women are only allowed to take the medication at an address in England where they permanently or usually reside. When the Abortion Act was passed in 1967, medical abortion did not exist and so when abortion medication came into use the 1967 law was *interpreted* as requiring the medication to be taken in a healthcare setting. Abortion legislation can be interpreted very broadly (McReynolds-Pérez 2017), and so in England, Scotland, and Wales, the Act was re-interpreted to bring the procedure in line with other countries such as France and Sweden and with World Health Organization recommendations. Testimonies such as the ones from Claudia and Paula were crucial to raising awareness of the unnecessarily traumatic journeys women were forced to take and to changing the law.

Similar testimonies emerge from the UK-Ireland abortion corridor. Again, women shared their experiences for political reasons during the 2018 campaign to change the law on abortion in the Republic of Ireland. While the earlier examples come from women traveling from the clinic to their home in the same country, Irish women who have sought abortions in the UK have to travel by plane or ferry, sometimes while going through an abortion. Sara, for example, had to travel from Dublin to Manchester and back in one day because she couldn’t afford a hotel. She told a reporter

[i]t was the worst thing, to have to get on a flight afterwards. I wanted to get into a clean bed and go to sleep. I didn’t want to be worrying about getting bags on and off a plane, and organising a taxi to get home. I was worried about what would happen if I got on to the flight and started bleeding uncontrollably. (Gentleman 2015, np).

A similar story came from Caoimhe Anglin, 28, who also travelled from Dublin to Manchester for an abortion. In her words,

[t]he travelling home was so difficult, you’re so tired and then you have to get on a train, go to the airport and then wait on the floor of Manchester airport in Terminal 3, which has no seats while you’re doubled-over in pain. It’s so difficult - it’s so stressful to get home (Scott 2017, np).

Lucy Watmough travelled from Ireland to London for an abortion and ‘on the packed train back to London’s Gatwick Airport, Watmough was forced to stand for over an hour, bleeding profusely and unable to ask her fellow passengers for a seat because there was no way of explaining she’d just had an abortion’ (McKenzie 2018, np). The reproductive health NGO, Marie Stopes, provides online advice about how to plan for traveling home to Ireland after an abortion in the UK. Their website urges women to ‘carefully **think about how you will manage possible bleeding** and pain during your flight home before you leave Marie Stopes UK’ (Marie Stopes n.d., np, emphasis in original). The fear of bleeding on the plane prompts some to take a more private option. Arlette Lyons travelled from Ireland to Liverpool by ferry for an abortion after learning that a chromosomal abnormality meant that her baby would not reach full term. She and her husband decided to drive and take the ferry as this would provide her with privacy in her grief and pain. Lyons noted that this was still a traumatic experience, saying ‘[t]he pain is just awful, and the only place you should be is in bed. You shouldn’t be getting in car, on a boat, in a car again’ (McKenzie 2018, np). These testimonies gave a voice to the thousands of Irish women who have made such journeys due to the prohibition of abortion in the Republic. While mobilities scholarship has often focused on bodies, it has shied away from the messy, embodied experiences of movement. A viapolitical perspective, looking at the unusual site of the vehicle, made the experience of bleeding apparent. By taking the theme of blood, it is possible to find a common narrative running through experiences of abortion travel, one that is rich with political potential.

The negative experiences of abortion journeys are emotional as well as embodied and fear is another common narrative. While the overwhelming majority of women do not regret their abortion (Rocca et al. 2015), the experience of having to travel can make women feel ‘banished’ (Kelly and Tuszynski 2016), in ‘reproductive exile’ (Inhorn and Patrizio 2009) or in ‘abortion exile’ (Singer 2019a). For Kelly and Tuszynski (2016, 26), women ‘ … are reminded at each step of their journey that they are undeserving of medical care at home’. As one anonymous woman who travelled from Ireland to the UK has explained, ‘[t]he scariest part was being in a different country with no support from people in Ireland … I felt so vulnerable. All this would have been less terrifying if I had been able to get this done in Ireland instead’ (Not At Home 2018, np). Likewise, in Mexico women can travel from states where abortions are not legal to Mexico City where they are legal. But the experience of travel, even within their own country, can be a scary experience. As one interviewee, Sofia Garduño Huerta from Fondo Maria, an organisation which helps women travel to Mexico City for abortions, explained, if the journey the woman has to take to Mexico City is over 7 or 8 hours, they will try to book her onto a flight but ‘there are some who have never been on a plane before, so that’s another fear’. Then it may be their first time in the capital so ‘there are several levels of fear, of the procedure, of what will happen, but also of leaving where they’re from, how to get to the city, how to get around it’. Women are also often negotiating patriarchal control from their partner or family with the fear of being caught. Similarly, Singer’s (2019a) work on women who travel from other states to Mexico City for a legal abortion highlights the multiple obstacles they face. Practicalities around travel time, available transport, and childcare affected how travel is experienced, if they were able to travel at all. For some women, traveling to the capital is a routine part of their lives while for those traveling from farther afield the trip is stressful and anxiety-inducing. It is poorer women and those from smaller, rural communities who disproportionately encounter these obstacles.

Fear is clearly present even when traveling for a legal abortion, but it is exacerbated when abortion is illegal and unsafe. Through my research on Chile-Peru abortion crossings, fear was evident. Abortions in both Chile and Peru are illegal in almost every circumstance but Chilean women travel to Peru where abortions are easier to access, cheaper, and more anonymous. Women travel to clandestine clinics that are of varying hygiene and ethical standards. In interviews with health workers, I was told that they are ‘scary places’ and that ‘they don’t have the best conditions’. One young woman who had travelled from Chile to Peru for an abortion told me ‘obviously I was scared for my life, oh it was awful, yeah I was terrified, it’s like these are things that aren’t legal you know, anything illegal is going to scare you because it’s not safe, you can’t go to someone if it goes wrong, you don’t have that right.’ This woman knew that her abortion was more likely to be unsafe and was terrified that it would be reported to the authorities. These journeys are therefore imbued with an emotional politics of fear and the lens of viapolitics allows us to focus on how these journeys are experienced.

What the above testimonies and experiences show is the necessity to take the journeys and vehicles of abortion transport seriously in terms of the emotional toll and traumatic embodied experience they entail for women. While the possibility of at least being able to manage to have access to travel for an abortion is emancipatory for the women to an extent, the realities of what these women endure is far from it. While wealthier women are able to travel to a safe doctor or even experience the privilege of fixity by paying for a local clandestine doctor, poorer women are more likely to have to visit an unsafe ‘clinic’ or travel while still bleeding. By focusing on the realities of abortion journeys, particularly here on the experience of bleeding en route and the fear women feel, the politics of abortion access and its mediation on the journey comes to the fore. Viapolitics is a way to pay witness to the emotional and embodied experiences of abortion travel by focussing on the route, the in-between, and the vehicles used.

## Politicising the vehicle

Vehicles do not only transport women for abortions. In taking vehicles seriously it is possible to explore how they themselves are sites of power and contestation and can be used for strategic political action. For Walters (2015a), it is important to examine ‘how certain images of vehicles function as visual operators’, and how the visual can be used strategically. In his work on how activists have protested deportation flights, for example, Walters (2015b) recounts how the activists mimicked the Lufthansa branding. Just as with migration politics, abortion activism also uses the visual power of vehicles in strategically political ways. I consider this is to be the spectacle of abortion activism. ‘Spectacle’ is used to create a reaction, often one that it is knowingly divisive (Kershaw 2003) and ‘provide[s] an element that transcends local conditions and the interconnections of day-to-day operations’ (Ponzini 2019, 80). Vehicles have been used as direct-action activism for other political goals. In this journal, Furness (2007) examines the mobilisations of Critical Mass activists who assemble on bikes in their thousands to contest the domination of ‘automobility’ of urban space and bikes have more recently been used by Deliveroo workers staging a mass bike ride protest to demand better working conditions. Pro-choice activists likewise engage in spectacular activism using vehicles in order to raise awareness and influence change, often in controversial ways. As I show here, through employing boats, trains, buses, and convoys, vehicles have become sites of abortion activism.

Consider Women on Waves, a Dutch pro-choice organisation founded in 1999 by the physician Rebecca Gomperts. They are infamous for their activist campaigns to contest barriers to abortion access and particularly for their ‘abortion boats’ which have been labelled as ‘one of the most audacious instances of feminist activism in recent memory’ (Lambert-Beatty 2008, 309). Once their boats are 12 nautical miles offshore from any country, they fall under Dutch law due to the Dutch flag they fly, and so Women on Waves can provide medical abortions at sea without flouting the laws of the country where they picked up the women on board (Gomperts 2002). While few women in practice receive an abortion on these boats, the campaigns are highly photogenic and provocative and therefore create a significant amount of media attention. It is through creating a spectacle with an audacious and captivating stunt, knowing that these images will be shared in the media, that the boat campaigns function. Gomperts has an art degree as well as a medical one and is well versed in the political possibilities of activist art (Lambert-Beatty 2008). In February 2017, Women on Waves visited Guatemala where the military refused to let the boat leave or re-enter Guatemalan waters under order from the President. This military intervention meant that the campaign was headline news in Guatemala and featured in the international press and calls to the safe abortion hotline increased which allowed activists to refer women who called to partner organisations. The images of Women on Waves activists locked on the pier and of the military escorting the ship into international waters were prime media fodder and their dissemination was what made the campaign successful. Their abortion boat campaigns have taken place in locations across the world and are viapolitical in the way the vehicle becomes centred as a political spectacle.

A second way in which abortion activists have politicised the vehicle is through the train. In May 1971 Irish activists transported then-banned condoms and contraceptive jellies by train from Belfast to Dublin which they showed to the customs officials at Connolly Train Station, who chose not to act (Murphy-Lawless and McCarthy 1999). In October 2014, a new generation of activists recreated this journey by train, this time to transport abortion medication (Gilmartin and Kennedy 2019). While the repetition of this journey tells a convenient narrative of activism around women’s reproductive rights in Ireland, the practicalities of the spectacle are murkier. This is because abortion medication is not legally available in Northern Ireland, so the feasibility of transporting pills from Belfast to Dublin does not hold up. However, as with the abortion boat, that is inconsequential to the symbolism of the stunt. The campaign needed to create a link to past activism in Ireland, showcase the slippery mobility of medication, and create ‘visual operators’ that would be featured in the media. In this way, it worked. The event using the train created a symbolic spectacle that had political resonance beyond the practicalities of the campaign.

As well as boats and trains, buses are another vehicle that have been employed in pro-choice activism. The socialist feminist group ROSA worked with Women on Web (a sister organisation to Women on Waves) in 2015 to travel by bus to Galway, Limerick, Cork, and Dublin in the Republic of Ireland (Gilmartin and Kennedy 2019). Women were able to visit the bus to receive phone consultations with medical professionals, which directly aided over 30 women, but the bus was also used to disseminate information about abortion pills more widely. The bus was used again in 2017 as the ‘Bus4Repeal’ in the run up to the Irish referendum on whether to repeal the 8^th^ amendment that effectively prohibited abortions by giving a foetus the same rights as the person carrying the foetus (Silver 2017). The bus as a site of political action has two benefits here. First, the flexible mobility of buses and roads means that the bus can travel to a great number of places; passing through villages and countryside as it travels between towns and cities. Second, the expansive sides of buses can act as a canvas for political messages. For example, a banner reading ‘safe abortion with pills’ was draped along the side of the ‘Bus4Repeal’ to be read as the bus travelled across Ireland, again a clear ‘visual operator’ (Walters 2015a). This is a more subtle spectacle but through these two ways, the political message and activism of the bus can be mobile as it travels.

Walters’ concept of viapolitics is important in not treating ‘the vehicle’ as an isolated and singular actor. We must always consider how vehicles work in relation to infrastructure, the environment, other vehicles, and other actors. One activist strategy where this connectivity was very apparent was the Canadian Abortion Caravan of 1970. This was a convoy of vehicles that travelled from Vancouver to Ottawa calling for free abortion on demand and for the repeal of abortion laws (Palmer 2011). Organised by the Vancouver Women’s Caucus, the caravan began with a collection of cars led by a bus with a coffin strapped atop which was filled with coat hangers to represent those who have died from illegal abortions (Sethna and Hewitt 2009). This example highlights the incredibly emotional and provocative possibility of the spectacle. The coffin resonated with unvoiced experiences of suffering and trauma. Each day, the caravan would drive further toward their destination, perform guerrilla theatre, and hold public meetings. On their arrival in Ottawa, approximately 300 activists, predominantly young women, marched on Parliament Hill, calling for a change to Canada’s abortion laws (Sethna and Hewitt 2009). There was a power to their symbolism and the vehicles moving together as a collective meant that they were able to gain political momentum.

Vehicles are not just a backdrop to abortion travel. As Walters (2015a) has argued in his case for viapolitics, ‘it is important to analyse vehicles as mobile sites of power and contestation in their own right’. As I have shown here, vehicles such as boats, trains, and buses are sites of political action where debates over abortion politics are played out. Vehicles are not just a medium to transport women for abortions, they can be used as agents themselves in struggles over abortion access. The vehicle as a political agent manages to cut through scale. These examples bring together state and international law with the individual experiences of needing abortion provision, which is what these activist campaigns are fighting for. As the four examples illustrate, the visual power of the vehicles themselves and the images that are shared are key to the contestation of abortion prohibition. Through creating a controversial spectacle, activists are able to grab the attention of the media, public, and politicians. This juxtaposes the aforementioned abortion journeys that are so often hidden and invisibilized. While individual examples of vehicles being used as sites of political action have been written about elsewhere (Sethna and Hewitt 2009; Gilmartin and Kennedy 2019; Lambert-Beatty 2008), there has not been a sustained theoretical engagement across these events. I advocate viapolitics as a conceptual framework for further engagement with vehicles in abortion scholarship but it also has rich potential for analyzing the political potential of vehicles in mobilities scholarship more broadly.

## Eliminating the vehicle

Abortion travel, even when made easier or cheaper, is still travel. As the earlier examples showed, travel can be a humiliating and traumatising experience and disproportionately impacts the poorest and most vulnerable. In abortion scholarship, immobility can be understood as the constraining or restriction of movement (Murray and Khan 2020), but fixity can also be the goal so that women need not travel at all (Side 2020). Mobility is not always synchronized with freedom. The possibility of fixity has emerged through the transportation of abortion medication. Commonly used for early pregnancies, medication has drastically changed how women access abortion (Calkin and Freeman 2018). In this section, I propose the notion of an ‘emancipatory viapolitics’, that is, one that renders the journey and the vehicles obsolete altogether. I will use abortion medication as an example to make the case for an emancipatory viapolitics that aims to avoid the traumatic and humiliating experiences associated with abortion travel.

Medical abortions are most commonly induced with the combination of the drugs mifepristone and misoprostol. They are used in the first trimester of pregnancy, are very safe, have efficacy rates of 95–98%, and the results are indistinguishable from a spontaneous miscarriage (Gomperts et al. 2008). They have been celebrated for giving pregnant people increased autonomy (Berer and Hoggart 2018) and for ‘de-medicalizing’ abortion (Manríquez et al. 2018). This creates a shift in who the ‘provider’ is and who ‘performs’ the abortion (Jelinska and Yanow 2018). In restrictive regimes these medications have made safe abortions possible while in more liberal regimes they provide an alternative for those who feel uncomfortable accessing care in a medical setting or are unable to. Medical abortion is particularly beneficial for people in rural areas as it increases their access to healthcare (Jones and Jerman 2013) and can contribute to decreasing maternal mortality and morbidity rates from unsafe abortions (Gomperts et al. 2008).With abortion only legal with the permission of a doctor in many countries, including the UK, activists have called for a de-medicalized and less paternalistic approach to abortion. With accurate information and support, pregnant people are able to take control of the process and clandestine abortions have become far safer.

Mifepristone and misoprostol have been on the list of essential medicines of the World Health Organization since 2005 but are only available clandestinely in many parts of the world (Gomperts et al. 2008). Misoprostol is also used to treat stomach ulcers and can therefore be obtained under false pretences while mifepristone which is solely used to end pregnancies is more challenging to procure. The increasing availability of abortion medication has had a dramatic impact on abortion travel. Whereas in 2001, 6,672 registered an address in Ireland when procuring an abortion in England, this number was almost halved by 2015 (Sheldon 2016). Aiken et al. (2018), in their study of women in Britain requesting abortion medication from the organisation Women on Web, found that 49% of all reasons given for using the service was barriers to access. Britain, despite having relatively liberal abortion legislation, still has barriers over eligibility for services, waiting times, distance to clinic, and other commitments that prevent travel. This means that abortion scholars need to reconsider how we think about the geography of abortion access and the mobilities involved.

One key way to do this is to explore how pills move. Once again, it is activist networks that are key to the transportation of these pills. These are often transnational flows with abortion pills bought online from one country and then transported through postal services to the woman (Sethna and Davis 2019). One of the largest organisations who facilitate this transportation is Women on Web. Women on Web was created as a sister organisation to Women on Waves due to requests for abortions on ‘the abortion boat’ from women. They provide information on abortion medication, how to self-induce an abortion with medication, and transport the pills to women, making abortion pills ‘readily accessible via a few clicks of a mouse’ (Sheldon 2016, 90). However, it is important to note that being able to order pills online does not remove the need for travel completely. Some organisations refuse to post pills directly to addresses in the Republic of Ireland because supplying medicines by mail order is illegal and customs officials can seize the pills (Fletcher 2014). One workaround is to arrange for the pills to be sent to Northern Ireland and travel there to collect them in person. Yet, in most locations, pills can be sent directly to the individual to use.

There is an important spatiality to how pills and knowledge of how to safely take them is disseminated. In locations where self-administering these medications is illegal, information about the services are shared on social media, on the streets, and through networks (Bloomer, Pierson, and Estrada Claudio 2019). In Mexico, pill-sharing networks exist in a small-scale, ad hoc way. Multiple abortion activists I have interviewed in Mexico explained how the medication misoprostol is sold in a box with more pills than are needed for an abortion. As misoprostol is used for the treatment of gastric ulcers it is legal and available for purchase over the counter throughout Mexico (Singer 2019b). Women are encouraged to hold on to the extra pills to pass on to someone they know or someone who an activist organisation will put them in touch with. One autonomist feminist group based on Mexico’s northern border who accompany women to self-abort with pills support women in this way. As they explained to me, ‘any girl who bought the pills and had some left over, we ask them to donate those pills to other girls, not to give them to us, but to give them to another girl. We want to create a network between girls’. Another activist, a woman of a post-menopausal age, told me how she buys misoprostol from the pharmacy knowing the pharmacist would not suspect her using them for an abortion given her age. As she said; ‘me, for example, I’m 51 and I have been [to buy misoprostol from a pharmacy], but if the youngest in our network goes, who is 21, they won’t sell to her, we have tried it. But if I go an hour later they sell me the boxes I ask for’. She is then able to pass them on to those who need them. This pill sharing means that women do not have to take the often long, arduous journey to Mexico City where they would be able to access an abortion legally.

The transportation of these pills has not only been to routinely deliver medication to women so that they can self-induce an abortion. There has also been pro-choice activism that has transported pills in innovative ways to raise awareness of limits to abortion access. Like with the four examples in the previous section, this once again shows the visual power of the spectacle. This has most notably been seen with Women on Waves’ abortion drone and abortion robot. These two technologies of mobility are ripe for media attention. The abortion drone was first flown in 2015 carrying abortion medication from Frankfurt an der Oder in Germany to Slubice in Poland where two women then took the pills (Women on Waves 2015). Drone equipment was confiscated by the German police but after an investigation, charges were dropped. On 21 June 2016, the drone flew again from the Republic of Ireland to Northern Ireland where two women took the pills that were aerially transported across the border at Narrow Waters Castle (Women on Waves 2016). These stunts are primarily about awareness raising rather than abortion provision and also highlight the fact that even in restrictive circumstances, strategies of resistance will contest barriers to access with these strategies being effected through the mobility of vehicles such as drones. As Gomperts stated about the Irish drone, ‘[t]he point of today’s flight is to show that no matter whether abortion is illegal in Northern Ireland or the Republic of Ireland, women will access abortion pills’ (Vinograd 2016, np).

Women on Waves teamed up with the Irish activist group ROSA for another form of medication transportation in May 2018, this time using robots. A small robot delivered a package to a woman outside Belfast’s High Court, she took a pill from the package, and openly took it (Norkin 2018). Women on Waves declared that this was legal because the robot was being operated from the Netherlands (Women on Waves 2018). Gomperts told the press that ‘[w]e are helping women, but we are trying to do that within the law. We are using innovative technology like robots to show how ridiculous these laws are, because there’s different ways to overcome them’ (Leonard 2018, np). This action was supported by Solidarity TD Ruth Coppinger who said ‘[t[hese acts of civil disobedience supplied pregnant people with safe but illegal abortion pills, but they were also instrumental in shifting the debate on abortion’ (Demolder 2018, np). Such stunts have a visual power and are effective at gaining media attention but they are also parodying the law. By playing with legal loopholes, the activists are able to bring new possibilities of abortion access into existence.

Whether supplying women with pills through the postal service and activist networks or performancebased activism to raise awareness of barrier to abortion access, these examples illustrate an emancipatory possibility. The transportation of medication eliminates (in many cases) the need for women themselves to travel. This reduces the burden of time, cost, and trauma that abortion travel has so often brought. Women will still experience bleeding, but this can be in the privacy of their own home rather than on a bus, train, or aeroplane. This is emancipation with caveats, then: women may not have a *right* to control their own reproductivity but, in a relative sense, they are *more* in control than they were. Taking control of the abortion process at home is emancipatory in the circumstances even if it cannot be a complete liberation. The greatest emancipatory potential here is in how the transportation of pills provides an option for women who cannot travel due to their citizenship or visa status. As Bloomer, Pierson, and Estrada Claudio (2019) have illustrated in the Republic of Ireland, asylum seekers and undocumented migrants face extra barriers to international travel, making the journey for an abortion in the UK or elsewhere incredibly difficult or impossible. This does not mean that accessing abortion pills is always straightforward; it relies on knowledge that they exist, access to a telephone or the internet, and a way to receive them. However, for a great number of people around the world, abortion pills can be understood as ‘emancipatory viapolitics’ whereby the journeys of ‘things’ reduces the burden of travel.

## Conclusion

This paper has offered a new understanding of abortion journeys, how they are experienced, and how vehicles are a part of this. Through a grounding in case studies from Latin America, North America, and Europe this paper has made three theoretical contributions: it has furthered the idea of ‘abortion mobilities’; brought the concept of viapolitics into mobilities scholarship; and proposed the term ‘emancipatory viapolitics’.

‘Abortion mobilities’ are the movement and fixity of people and things that shape abortion access. Within the ‘new mobilities paradigm’ both reproduction and gender remain underdeveloped (Clarsen 2014; Speier, Lozanski, and Frohlick 2020) and in this paper I have explained how a focus on abortion is politically vital and central to the concerns of mobility studies. In order to do this I used the concept of ‘viapolitics’. For Walters (2015a), viapolitics represents an underutilised perspective, one where the interaction between people and vehicles is an important lens on migratory struggles. I believe it is possible to swap ‘migratory’ for ‘abortion’ here. Travel and transport have underpinned struggles over abortion access for women across the world and abortion scholars should therefore pay greater attention to the material, emotional, and embodied realities of abortion mobilities. In studying abortion politics from the perspective of the journey and the vehicle, it is possible to better understand how power works and what the possibilities for political action and social justice are. The ‘vehicle’ cuts through abortion politics in two important ways: in how women travel for abortions and as sites where activists protest barriers to abortion access. Vehicles are also notable for their absence in changing patterns of access whereby abortion pills travel to women, rather than women travelling to clinics. William Walters’ concept of viapolitics is relevant to scholarship on abortion travel because it shifts the focus to the angle of the vehicle and the journey. This places increased emphasis on *how* abortions are accessed and how prohibition is resisted. Such a form of abortion mobilities shows how the vehicle is entangled in abortion politics and is also mobilised in abortion activists’ calls for abortion to be free, safe, legal, and *local.*

Viapolitics served as a useful theoretical framework for me to develop the concept of abortion mobilities but it has the potential to enrich mobilities scholarship more generally. William Walters coined the term to provide a framework for centring vehicles, roads, and routes in theorising migration politics from the perspective of the in-between. This is conducive to a mobility studies that goes beyond movement as physical transportation to interrogate the politics and inequalities of mobilities (Sheller and Urry 2016). Viapolitics can invigorate the study of transport, particularly in how the concept does not treat ‘the vehicle’ as isolated or singular. Another contribution that viapolitics makes to mobilities scholarship is that it is not exclusively focused on people; instead it takes vehicles and infrastructure seriously and fosters a relational politics (Adey 2006). Therefore, the term viapolitics has only been used within migration politics scholarship but, as I have set out here, it has real potential to be employed in mobilities scholarship more broadly.

While viapolitics is useful in its current form, it can be stretched further and ‘emancipatory viapolitics’ is just one example of how this can be fruitful. In this paper I have furthered viapolitics through considering bodily immobility and the transportation of goods, in this case abortion medication, as a way to eliminate the need for the travel of women. I believe that this contribution can prompt innovative scholarship utilising viapolitics both within and beyond migration politics in two ways. Firstly, viapolitics could focus more explicitly on immobility and stasis. This would be useful within migration politics to examine slowing and stopping on migratory journeys such as detention, deportation, and camps. It would also allow for an understanding that migrants often have a desire for stasis, just as abortion travellers do. Secondly, it can prompt scholars to examine the mobilities of goods through the lens of viapolitics. It is not just people who are entangled with vehicles and infrastructures, things are too. As shown through the movement of abortion pills, the transportation of things can offer the privilege of stasis, or what I term ‘emancipatory viapolitics’.

This paper has therefore shown how the vehicle in the abortion journey provides a window into the politics of abortion mobilities. Viapolitics draws attention to the journey, the vehicles, the infrastructure of movement, and how these become entangled with relations of power, knowledge, and resistance. It is therefore a valuable analytical tool for interrogating the ‘in-between’ of abortion mobilities which largely remain overlooked in current scholarship on abortion but also mobilities more broadly. However, the case of abortion mobilities also brings to light the emancipatory potential of certain viapolitical interventions that seek to replace those vehicles that transport women with other vehicles that transport goods, removing the need for women to travel in the first place. Hence, as the case of abortion medication clearly demonstrates, there is also a need to move the discussion of viapolitics beyond people to things and also analyse how these are mobilised for emancipatory ends.

## Data Availability

Due to ethical concerns, the research data supporting this publication are not publicly available.

## Note

1 There are two main types of abortion: medical and surgical. Medical abortion involves taking medication (mifespristone and misoprostol) to end the pregnancy. Surgical abortion is a short procedure that uses vacuum aspiration for earlier pregnancies or dilation and evacuation for later ones (usually from 15 weeks onwards).

## References

[R1] Adey P (2006). If Mobility Is Everything Then It Is Nothing: Towards a Relational Politics of (Im)mobilities. Mobilities.

[R2] Aiken AR, Guthrie KA, Schellekens M, Trussell J, Gomperts R (2018). Barriers to Accessing Abortion Services and Perspectives on Using Mifepristone and Misoprostol at Home in Great Britain. Contraception.

[R3] Apostolides Z (2018). Abortion Pill Horror. The Sun.

[R4] BBC News (2018). Abortion Pill Can Be Taken at Home in England, under New Plan. BBC News.

[R5] Berer M, Hoggart L (2018). Medical Abortion Pills Have the Potential to Change Everything about Abortion. Contraception.

[R6] Bergmann S (2011). Fertility Tourism: Circumventive Routes that Enable Access to Reproductive Technologies and Substances. Signs: Journal of Women in Culture and Society.

[R7] Bialasiewicz L, Maessen E (2018). Scaling Rights: The ‘Turkey Deal’ and the Divided Geographies of European Responsibility. Patterns of Prejudice.

[R8] Bloomer F, Pierson C, Estrada Claudio S (2019). Reimagining Global Abortion Politics: A Social Justice Perspective.

[R9] Brown LA, Sethna C, Davis G (2019). Abortion across Borders: Transnational Travel and Access to Abortion Services.

[R10] Calkin S (2019). Towards a Political Geography of Abortion. Political Geography.

[R11] Calkin S, Freeman C (2018). Trails and Technology: Social and Cultural Geographies of Abortion Access. Social & Cultural Geography.

[R12] Clarsen G, Adey P, Bissell D, Hannam K, Merriman P, Sheller M (2014). The Routledge Handbook of Mobilities.

[R13] Craig C (2018). I Had to Risk Miscarrying in a Taxi after Taking an Abortion Pill. Women Should Be Allowed to Take It at Home. The Independent.

[R14] Cresswell T (2010). Towards a Politics of Mobility. Environment and Planning D: Society and Space.

[R15] Demolder K (2018). Pro-choice Activists Have Trained Robots to Deliver Abortion Pills to Northern Ireland. Joe.

[R16] Deomampo D (2013). Gendered Geographies of Reproductive Tourism. Gender & Society.

[R17] Doran F, Nancarrow S (2015). Barriers and Facilitators of Access to First-Trimester Abortion Services for Women in the Developed World: A Systematic Review. Journal of Family Planning and Reproductive Health Care.

[R18] Doran FM, Hornibrook J (2016). Barriers around Access to Abortion Experienced by Rural Women in New South Wales, Australia. Rural & Remote Health.

[R19] Fletcher R (2014). Contesting the Cruel Treatment of Abortion-seeking Women. Reproductive Health Matters.

[R20] Fortunati L, Taipale S (2017). Mobilities and the Network of Personal Technologies: Refining the Understanding of Mobility Structure. Telematics and Informatics.

[R21] Freeman C (2015). Violence on the Chile-Peru Border: Arica 1925-2015.

[R22] Freeman C (2017). The Crime of Choice: Abortion Border Crossings from Chile to Peru. Gender, Place & Culture.

[R23] Furness Z (2007). Critical Mass, Urban Space and Velomobility. Mobilities.

[R24] Gentleman A (2015). ‘It Was the Scariest Thing I’ve Ever Done’: The Irish Women Forced to Travel for Abortions. The Guardian.

[R25] Gilmartin M, Kennedy S, Sethna C, Davis G (2019). Abortion across Borders: Transnational Travel and Access to Abortion Services.

[R26] Gomez MM (2016). More than Mileage: The Preconditions of Travel and the Real Burdens of HB 2. Columbia Journal of Gender and Law.

[R27] Gomperts R (2002). Women on Waves: Where Next for the Abortion Boat?. Reproductive Health Matters.

[R28] Gomperts RJ, Jelinska K, Davies S, Gemzell-Danielsson K, Kleiverda G (2008). Using Telemedicine for Termination of Pregnancy with Mifepristone and Misoprostol in Settings Where There Is No Access to Safe Services. BJOG: An International Journal of Obstetrics & Gynaecology.

[R29] Hannam K, Sheller M, Urry J (2006). Mobilities, Immobilities and Moorings. Mobilities.

[R30] Heller C, Pezzani L, Stierl M (2017). Disobedient Sensing and Border Struggles at the Maritime Frontier of Europe. Spheres: Journal for Digital Cultures.

[R31] Inhorn MC, Patrizio P (2009). Rethinking Reproductive ‘Tourism’ as Reproductive ‘Exile’. Fertility and Sterility.

[R32] Inhorn MC, Patrizio P (2012). Procreative Tourism: Debating the Meaning of Cross-border Reproductive Care in the 21st Century. Expert Review of Obstetrics & Gynecology.

[R33] Jelinska K, Yanow S (2018). Putting Abortion Pills into Women’s Hands: Realizing the Full Potential of Medical Abortion. Contraception.

[R34] Jerman J, Frohwirth L, Kavanaugh ML, Blades N (2017). Barriers to Abortion Care and Their Consequences for Patients Traveling for Services: Qualitative Findings from Two States. Perspectives on Sexual and Reproductive Health.

[R35] Jones RK, Jerman J (2013). How Far Did U.S. Women Travel for Abortion Services in 2008?. Journal of Women’s Health.

[R36] Kelly L, Tuszynski N (2016). Banishing Women: The Law and Politics of Abortion Travel. Columbia Journal of Gender and Law.

[R37] Kershaw B (2003). Curiosity or Contempt: On Spectacle, the Human, and Activism. Theatre Journal.

[R38] Lambert-Beatty C (2008). Twelve Miles: Boundaries of the New Art/Activism. Signs: Journal of Women in Culture and Society.

[R39] Leonard V (2018). Three Women Take Abortion Pills at Pro-choice Rally outside Court. Belfast Telegraph.

[R40] Manríquez IP, Standen CM, Carimoney AÁ, Richards A (2018). Experience of Clandestine Use of Medical Abortion among University Students in Chile: A Qualitative Study. Contraception.

[R41] Marty R (2019). The Road to Abortion Is Paved with Bad Bus Routes. Talk Poverty.

[R42] McKenzie S (2018). Irish Abortions Happen; They Just Don’t Happen on Irish Soil. CNN.

[R43] McReynolds-Pérez J (2017). Abortion as Empowerment: Reproductive Rights Activism in a Legally Restricted Context. BMC Pregnancy and Childbirth.

[R44] Murphy-Lawless J, McCarthy J (1999). Social Policy and Fertility Change in Ireland: The Push to Legislate in Favour of Women’s Agency. European Journal of Women’s Studies.

[R45] Murray L, Khan N (2020). The Im/Mobilities of ‘Sometimes-migrating’ for Abortion: Ireland to Great Britain. Mobilities.

[R46] Norkin L (2018). Activists Used ‘Abortion Robots’ to Deliver Pills to Women in Northern Ireland. Glamour.

[R47] Not At Home (2018). What It’s like to Travel to a Foreign Country for an Abortion. Vice.

[R48] Palmer B (2011). ‘Lonely, Tragic, but Legally Necessary Pilgrimages’: Transnational Abortion Travel in the 1970s. Canadian Historical Review.

[R49] Pheterson G, Azize Y (2005). Abortion Practice in the Northeast Caribbean: ‘Just Write down Stomach Pain’. Reproductive Health Matters.

[R50] Ponzini D, Molotch H, Ponzini D (2019). The New Arab Urban: Gulf Cities of Wealth, Ambition, and Distress.

[R51] Pruitt L, Vanegas M (2015). Urbanoramativity, Spatial Privilege and Judicial Blind Spots in Abortion Law. Berkeley Journal of Gender, Law and Justice.

[R52] Rocca CH, Kimport K, Roberts SC, Gould H, Neuhaus J, Foster DG (2015). Decision Rightness and Emotional Responses to Abortion in the United States: A Longitudinal Study. PLoS One.

[R53] Scott C (2017). Irish Woman Who Was Left ‘Bleeding Heavily’ after Abortion Was Afraid of Going to an Irish Doctor. DublinLive.

[R54] Senu A, Walters W, Heller C, Pezzani L Viapolitics: Border, Migration, and the Power of Locomotion.

[R55] Sethna C, Davis G (2019). Abortion across Borders: Transnational Travel and Access to Abortion Services.

[R56] Sethna C, Doull M (2012). Accidental Tourists: Canadian Women, Abortion Tourism, and Travel. Women’s Studies.

[R57] Sethna C, Hewitt S (2009). Clandestine Operations: The Vancouver Women’s Caucus, the Abortion Caravan, and the RCMP. Canadian Historical Review.

[R58] Sheldon S (2016). How Can a State Control Swallowing? The Home Use of Abortion Pills in Ireland. Reproductive Health Matters.

[R59] Sheller M (2020). The Reproduction of Reproduction: Theorizing Reproductive (Im)mobilities. Mobilities.

[R60] Sheller M, Urry J (2016). Mobilizing the New Mobilities Paradigm. Applied Mobilities.

[R61] Side K (2020). Abortion Im/mobility: Spatial Consequences in the Republic of Ireland. Feminist Review.

[R62] Silva M, McNeill R (2008). Geographical Access to Termination of Pregnancy Services in New Zealand. Australian and New Zealand Journal of Public Health.

[R63] Silver L (2017). This Bus Is Driving around Ireland to Help Women Access Safe Abortion. Buzzfeed News.

[R64] Singer EO (2019a). Abortion Exile: Navigating Mexico’s Fractured Abortion Landscape. Culture, Health & Sexuality.

[R65] Singer EO (2019b). Realizing Abortion Rights at the Margins of Legality in Mexico. Medical Anthropology.

[R66] Speier A, Lozanski K, Frohlick S (2020). Reproductive Mobilities. Mobilities.

[R67] Statz M, Pruitt LR (2019). To Recognize the Tyranny of Distance: A Spatial Reading of Whole Woman’s Health V. Hellerstedt. Environment and Planning A: Economy and Space.

[R68] Stopes M Flying Back to Ireland after Abortion.

[R69] Teunissen P (2018). Border Crossing Assemblages: Differentiated Travelers and the Viapolitics of FlixBus. Journal of Borderlands Studies.

[R70] Uteng TP, Cresswell T (2008). Gendered Mobilities.

[R71] Vinograd C (2016). Abortion Drone from Women on Web Delivers Pills to Northern Ireland. NDC News.

[R72] Walters W (2015a). Migration, Vehicles, and Politics: Three Theses on Viapolitics. European Journal of Social Theory.

[R73] Walters W, Fuggle S, Lanci Y, Tazzioli M (2015b). Foucault and the History of Our Present.

[R74] Walters W (2016). The Flight of the Deported: Aircraft, Deportation, and Politics. Geopolitics.

[R75] Women on Waves (2015). First Flight Abortion Drone, Poland 2015.

[R76] Women on Waves (2016). Abortion Drone Ireland, 2016.

[R77] Women on Waves (2018). Abortion Robots Will Deliver Abortion Pills in Belfast, Northern Ireland.

[R78] Yeoh BS, Ramdas K (2014). Gender, Migration, Mobility and Transnationalism. Gender, Place & Culture.

